# Using Different Types of Artificial Neural Networks to Classify 2D Matrix Codes and Their Rotations—A Comparative Study

**DOI:** 10.3390/jimaging9090188

**Published:** 2023-09-18

**Authors:** Ladislav Karrach, Elena Pivarčiová

**Affiliations:** Department of Manufacturing and Automation Technology, Faculty of Technology, Technical University in Zvolen, Masarykova 24, 960 01 Zvolen, Slovakia; karrach@zoznam.sk

**Keywords:** multilayer perceptron, probabilistic neural network, radial basis function neural network, convolutional neural network, 2D matrix codes, classification

## Abstract

Artificial neural networks can solve various tasks in computer vision, such as image classification, object detection, and general recognition. Our comparative study deals with four types of artificial neural networks—multilayer perceptrons, probabilistic neural networks, radial basis function neural networks, and convolutional neural networks—and investigates their ability to classify 2D matrix codes (Data Matrix codes, QR codes, and Aztec codes) as well as their rotation. The paper presents the basic building blocks of these artificial neural networks and their architecture and compares the classification accuracy of 2D matrix codes under different configurations of these neural networks. A dataset of 3000 synthetic code samples was used to train and test the neural networks. When the neural networks were trained on the full dataset, the convolutional neural network showed its superiority, followed by the RBF neural network and the multilayer perceptron.

## 1. Introduction

Two-dimensional matrix codes are built of dark and light modules, usually arranged in a square matrix. The size of this matrix depends on the amount of data to be encoded in the matrix code (as the amount of data increases, the dimensions of the matrix increase).

Each type of two-dimensional matrix code has its characteristic fixed parts, which are common to all matrix codes of the given type and allow different types of matrix codes to be distinguished from each other. These fixed parts, which serve to locate the code and determine its orientation, are called finder patterns.

**Figure 1:**
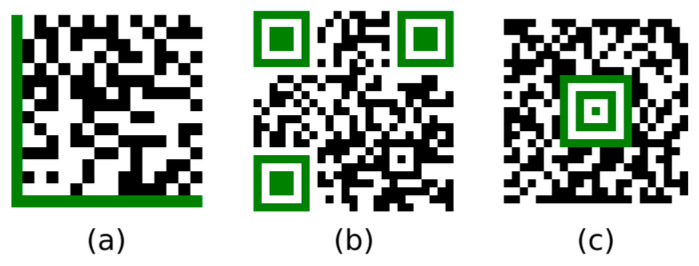
Two-dimensional matrix codes, (**a**) Data Matrix code, (**b**) QR code, (**c**) Aztec code.

The most well-known and commonly used types of matrix codes are Data Matrix codes, QR codes, and Aztec codes (Figure 1). Each of these three matrix codes has its own characteristic finder patterns (Data Matrix code: “L” pattern on two adjacent sides; QR code: inner dark square in a dark frame on three corners; Aztec code: “bullseye” in the centre).

These 2D matrix codes can encode thousands of alphanumeric characters and include an error correction feature so that the stored data can still be decoded if the code is partially damaged. As a key component of automatic identification and data capture technology, 2D matrix codes are often applied to parts in manufacturing, transport units in logistics, warehouse locations, sold goods, posters, business cards, tickets, etc. Computer vision applications must therefore be able to distinguish these codes in order to decode them correctly.

In the following text, we present four types of artificial neural networks (ANNs) and evaluate their ability to classify these three types of 2D matrix codes. We are only concerned with the classification of codes that have already been localised. The issue of localisation and recognition of 2D matrix codes in arbitrary images was discussed in [1,2]. In addition to classifying the type of 2D matrix code, we also deal with the issue of using ANNs to determine the orientation (rotation) of the 2D matrix code. This is because correct decoding of the code requires the code to be oriented in its base (non-rotated) position (as shown in Figure 1).

### Related Work

Several authors address the localisation and recognition of one typeof 2D matrix codes in images. One group of methods is based on the extraction of features from a sliding window and their subsequent classification (i.e., determining whether it is part of a matrix code or not). Smaller adjacent regions identified as part of the matrix code are then merged into larger regions. Another set of methods identifies the matrix code in the image as a whole.

Bodnár and Nyúl [3] trained six weak classifiers using Haar-like features, local binary patterns, and histograms of oriented gradients (as implemented in the OpenCV library). These features were extracted from the finder patterns and from the entire QR code. The sample size was 32 × 32. Gaur and Tiwari [4] extracted statistical features (mean, standard deviation, smoothness, skewness, uniformity, and entropy) from non-overlapping 80 × 70 px image blocks and used the MLP neural network to determine whether the blocks were part of a QR code or not. Grósz et al. [5] experimented with a feed-forward neural network with one and three hidden layers and with sigmoid and ReLU activation functions. The input feature vector was extracted from an edge magnitude map from a circular pattern of overlapping blocks of a predefined size.

In the first phase of their research, Hansen et al. [6] utilized the YOLO object detection algorithm (based on the Darknet-19 CNN architecture) to detect 1D and QR codes in a whole image, while in the second phase, another angle prediction network (also based on Darknet-19) was used. Almeida et al. [7] investigated different types of object detectors (Faster R-CNN, SSD, YOLO) based on CNNs to locate Data Matrix codes used as navigation landmarks. YOLOv4 was found to be the best detector, followed by a conventional decoder (libdmtx). Che et al. [8] trained an eight-layer CNN to identify the type of distortion and quality grade of industrial Data Matrix codes. Chou et al. [9] proposed an algorithm to localise and segment QR codes, also using a convolutional neural network.

Huo et al. [10] used a back-propagation neural network to correct the distortion of QR codes. Waziry et al. [11] studied the performance of different CNN models for noise type classification in QR codes.

All of the above works deal with only one type of 2D matrix code (most often with QR codes). In our article, we use ANNs to distinguish between images of different types of matrix codes (namely Data Matrix codes, QR codes, and Aztec codes).

## 2. Materials and Methods

All four types of artificial neural networks (ANNs), which will be presented in turn, have an image in the input layer (the number of neurons in the input layer is equal to the number of points in the image) and five neurons in the output layer, where each neuron corresponds to one of the five classes into which the images are to be classified (1—Data Matrix code, 2—QR code, 3—Aztec code, 4—Code 128 (1D barcode), 5—Characters (non-barcode objects)). 1D barcodes and text have been added to matrix codes because they often appear together, for example, on labels or posters.

### 2.1. Multilayer Perceptron (MLP)

**Figure 2:**
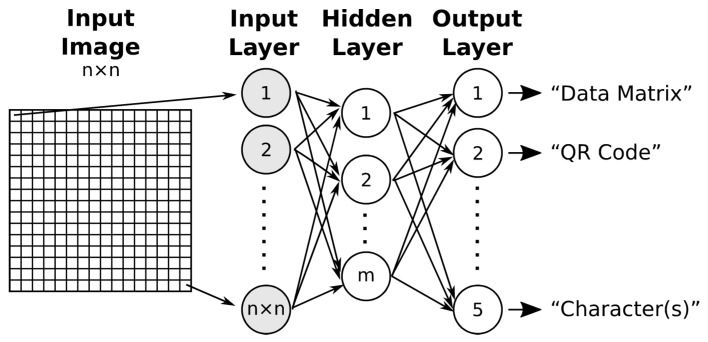
Scheme of a two-layer feed-forward neural network.

A multilayer perceptron is a basic type of feed-forward ANN [12]. A neural network consists of an input layer, one or more hidden layers, and an output layer. Neurons in one layer are fully connected to neurons in the next layer (Figure 2). The number of the input neurons is equal to the number of elements in the feature vector (if the entire image is used directly as the feature vector, then the size of the feature vector is equal to the number of points in the image). The number of output neurons corresponds to the number of classes into which the images are to be classified.

**Figure 3:**
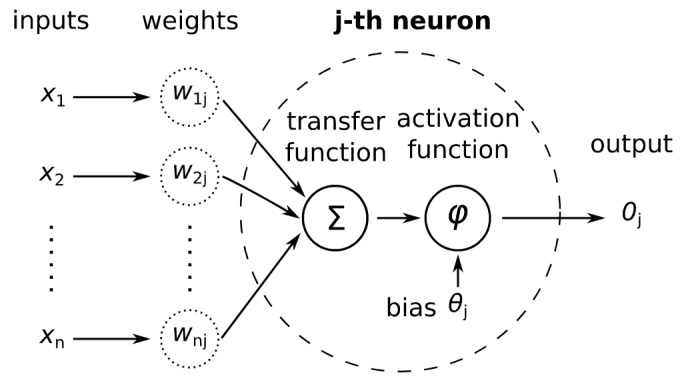
Scheme of an artificial neuron.

The neurons in the hidden and output layers compute a weighted sum of their inputs. The computed weighted sum is the input to the neuron’s activation function (Equation (1), Figure 3).

(1) $$o_{j}=\varphi \left(\sum_{i=1}^{n}x_{i}w_{ij}-\theta_{j}\right)$$

**Figure 4:**
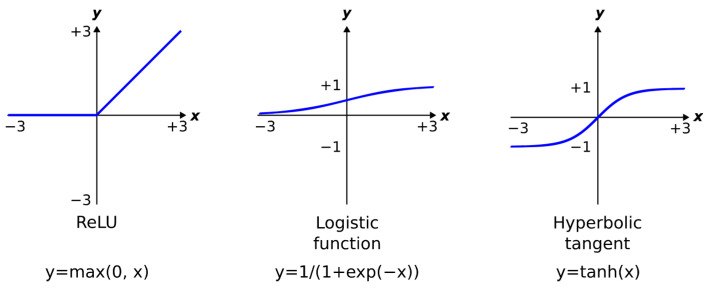
Common neuron activation functions.

Except for the input layer, the neurons have a non-linear activation function (typically a hyperbolic tangent or sigmoid function (Figure 4)); non-linear activation functions are required if the ANN is to learn complex data and correctly discriminate between classes separated by a non-linear decision boundary).

The ANN is trained using supervised learning and an error back-propagation algorithm to minimise the total error of the whole output [13].

As the size and number of layers in an ANN network increases, its capacity increases. A single hidden layer ANN is capable of universal approximation. The universal approximation theorem states that a feed-forward network with a single hidden layer containing a finite number of neurons can approximate continuous functions with mild assumptions by the activation function [14,15,16]. An ANN with two hidden layers can represent an arbitrary decision boundary to arbitrary accuracy with rational activation functions and can approximate any smooth mapping to any accuracy.

### 2.2. Probabilistic Neural Network (PNN)

**Figure 5:**
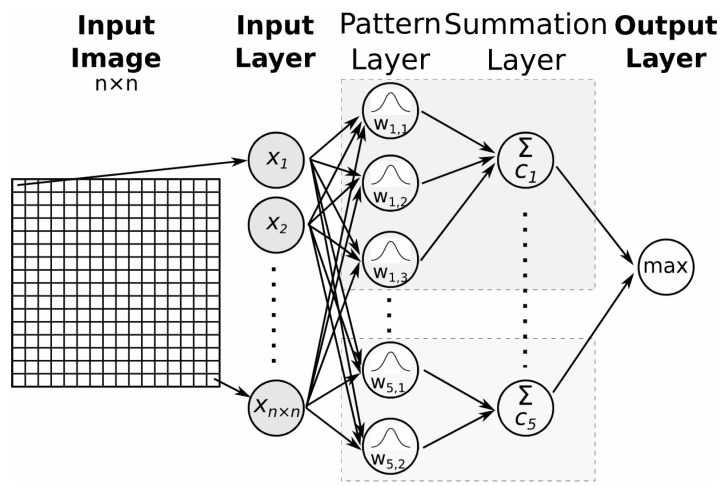
Scheme of a probabilistic neural network.

A probabilistic neural network is a type of feed-forward ANN with four layers (input layer, pattern layer, summation layer, and output layer). The number of neurons in the input layer is equal to the size of the feature vector. The input layer is fully connected to the first hidden layer (Figure 5).

The neurons of the first hidden layer—the pattern layer—are organised into groups, where one group represents one class. The number of neurons in the pattern layer is equal to the number of training samples. Synapses leading from the input layer to one pattern layer neuron store the feature vector values of one training sample. The pattern layer computes the Euclidean distance between the tested input vector and the input vectors from the training samples and applies the radial basis kernel function (Equation (2)), and its output is a vector expressing how close the tested input is to the inputs from the training samples.

(2) $${o1}_{ji}=\frac{1}{{\left(\sqrt{2\pi \sigma^{2}}\right)}^{n}}e^{\frac{-{\left(X-W_{ji}\right)}^{T}\left(X-W_{ji}\right)}{{2\sigma }^{2}}},$$

Here, *o*1 is the output from the pattern layer, *n* is the number of input neurons (size of the feature vector), *X* is the feature vector of the tested sample, *W*_ji_ is the *i*-th training sample of class *j*, *σ* is the smoothing parameter, and *T* is the transpose operator.

The neurons of one group (class) are connected to only one neuron in the second hidden layer—the summation layer. One neuron in the summation layer represents one class.

The number of neurons in the summation layer is equal to the number of classes. The summation layer neurons average the contributions from the pattern layer neurons belonging to the same class (Equation (3)).

(3) $${o2}_{j}=\frac{1}{n_{j}}\sum_{i=1}^{n_{j}}{o1}_{ji}$$

Here, *o2*_j_ is the output from the summation layer of the neuron representing class *j* and *n*_j_ is the number of training samples in class *j*.

The output of the summation layer is a probability vector. The class with the highest probability is selected in the output layer [17].

A probabilistic neural network has only one parameter—the smoothing parameter, *σ* (the spread value of the probability density function)—that needs to be “trained”. If *σ* is too small, the network will not be able to generalise; if it is too large, the network will not be able to discriminate between different classes. The value of the *σ* parameter can be:

- common to all pattern layer neurons (a cross-validation (between training and validation datasets) method that minimises network error can be used);
- common to pattern layer neurons belonging to the same class (the *σ* values can be calculated as half the average distance between the training samples in the same class or, for each training sample, it can be half the distance from that sample to the nearest other sample vector [18]);
- determined for individual features of the feature vector (standard deviation of training samples for each feature);
- determined for each class and feature of the feature vector.

### 2.3. Radial Basis Function Network (RBF NN)

**Figure 6:**
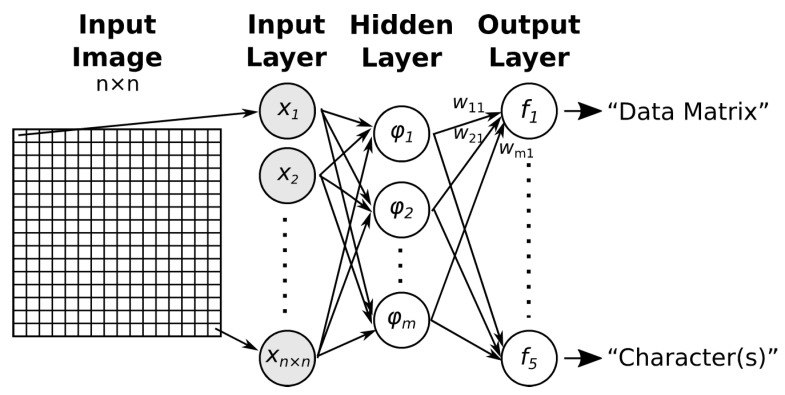
Scheme of an RBF neural network.

A radial basis function network (RBFN) typically has a three-layer structure [19]: an input layer, where the number of neurons is equal to the size of the feature vector, which is fully connected to the hidden layer; a hidden layer, where the neurons have a non-linear RBF activation function (usually a Gaussian function); and an output layer, where the neurons have a linear activation function (Figure 6).

Each hidden layer neuron computes the degree of similarity between its input vector and its prototype (central) vector (which is derived from the training set). The closer the input vector is to the prototype vector, the closer the value of the RBF function is to one (Equation (4)). The number of neurons in the hidden layer can be equal to the number of training samples, but is usually much smaller.

(4) $$\varphi_{i}\left(x\right)=e^{-\beta_{i}{\left\|X-\mu_{i}\right\|}^{2}},$$

Here, || is the Euclidean distance between the input vector *X* and the central vector *µ*_i_ of the i-th hidden layer neuron and *β*_i_ controls the width of the Gaussian curve.

The output layer performs a linear combination of the hidden layer outputs to obtain a final output probability (Equation (5)). Classification takes place only between the hidden layer and the output layer.

(5) $$f_{j}\left(x\right)=\sum_{i=1}^{m}w_{ij}\varphi_{i}(x)$$

Here, *f*_j_ is the output of the neuron representing class *j*, *m* is the number of neurons in the hidden layer, and *w*_ij_ is the weight from the i-th neuron in the hidden layer to the j-th neuron in the output layer.

The learning of RBF networks can be implemented as [20,21]:

- One-phase learning: central vectors are randomly selected from a set of input vectors (or all data points are used as central vectors), and typically a single predefined value for β is used. Then, only the weights of the output layer are adjusted by some method of supervised learning, e.g., minimizing the square of the differences between the network output and the desired output value;
- Two-phase learning: the hidden and output layers of the RBF network are trained separately. First, the centre’s µ_i_ and the scaling parameter’s β_i_ are determined. Then, the weights of the output layer are adjusted. A clustering algorithm such as K-Means can be used to select the centre’s µ_i_, while β_i_ is calculated as $\beta_{i}=1/2\sigma_{i}^{2}$, where σ_i_ is the average distance of the samples belonging to cluster i from the centre µ_i_;
- Three-phase learning: First, the RBF network is initialised using two-phase learning. Then, the entire network architecture is turned using another optimisation procedure.

### 2.4. Convolutional Neural Network (CNN or ConvNet)

**Figure 7:**
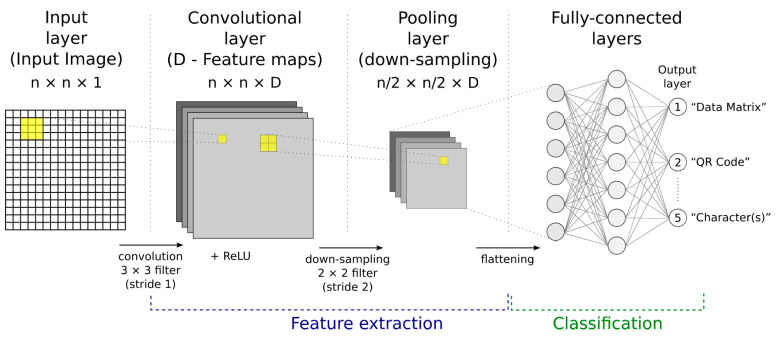
The basic building blocks of a convolutional neural network.

A convolutional neural network (CNN) is made up of several typical building blocks (layers) (Figure 7).

**Input layer (I)**: Unlike previous ANN types, a CNN explicitly assumes that the input is an image (an image represents spatially ordered data). The dimensions of the input image are often set to be multiply divisible by two, i.e., common sizes are 32, 64, 96, 224.

**Figure 8:**
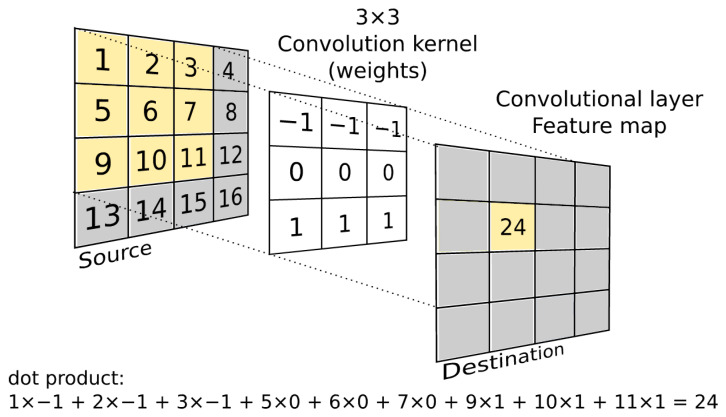
Convolution operation.

**Convolutional layer (C)**: Neurons in a convolutional layer are only connected to a small region (receptive field) of the previous layer, rather than all neurons in fully connected layers. Each convolutional layer neuron computes the dot product between its weights (mask, kernel, learnable filter) and the small region to which it is connected (Figure 8). Each convolutional layer works with multiple filters and creates multiple feature maps. The number of filters (as well as the number of feature maps created in the convolutional layer) determines the “depth” of the layer. The role of the different filters is to extract different features (the first layers capture low-level features such as corners, edges, endpoints, gradient orientation, colour; by increasing the number of convolutional layers, high-level features begin to be captured). Filters (convolutional kernels) are trained using a backpropagation algorithm (i.e., filters are not manually created, but their weights are randomly initialised and subsequently modified during training). Filters of size 3 × 3 and, for larger input images, 5 × 5 or even 7 × 7 are commonly used. A filter must always have the same number of channels as the input (often referred to as “depth”; the convolution operation uses a multi-channel kernel sliding over a multi-channel feature map to produce a single output feature map). The “stride” parameter determines the step by which the filter is moved along the input image (or along the feature map; both in the horizontal and in the vertical direction).

**Activation function**: An activation function is applied to the output of each convolutional layer. The non-linear rectified linear unit (ReLU) activation function is commonly used: *y* = max (0, *x*) (Figure 4).

**Figure 9:**
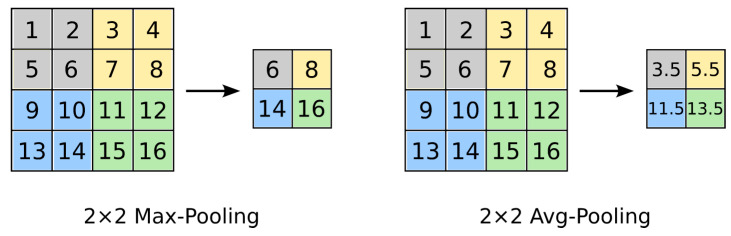
Pooling operation.

**Pooling layer (S)**: A pooling layer performs down-sampling and thus reduces the dimensionality (and computational complexity) of the network. A 2 × 2 patch (mask, kernel, filter), shifted with stride size of 2 over the input feature map, is commonly used (Figure 9). **Max. Pooling** calculates the maximum of the values under the path (highlights the most present feature under the patch), while **Avg. Pooling** calculates the average of the values under the patch.

One purpose of the pooling operation is to make the model independent of small differences in the positions of the extracted features (shift and distortion invariance), and the other is to reduce the amount of data for further processing layers, thereby speeding up the model. After several convolutional and pooling layers, the feature map sizes are reduced and more complex features are extracted. The output of the last convolutional or pooling layer is flattened and becomes the input of the fully connected layer.

**Fully connected layers (F)**: Every neuron in a fully connected (dense) layer is connected to every neuron in the previous layer. This is the same as a traditional multi-layer perceptron (MLP), which is simply an ANN with at least three layers [22].

The CNN architecture is determined by the way these building blocks (layers) are stacked and by their parameters, such as the number of feature maps, kernel size, and stride size [23,24,25,26,27]. Common CNN architectures are [28]:

- I → C + ReLU → F.
- I → [C + ReLU → S] × 2 → F + ReLU → F.
- I → [[C + ReLU] × 2 → S] × 3 → [F + ReLU] × 2 → F.
  (two convolutional layers (C) stacked before every pooling layer (S))

Where I is the input layer, C is the convolutional layer, ReLU is the non-linear activation function, S is the pooling layer, and F is the fully connected layer.

The training of the CNNs (as for the regular ANNs) consists of a forward pass and a backward pass. In the forward pass, the output of the CNN is calculated using the existing weights and biases (which are initialised with small random numbers at the start of training). In the backward pass, the error recorded at the output layer is fed back through the CNN and the weights and biases are updated to minimise the error (gradient descent back-propagation algorithm) [29]. Convolutional neural networks combine feature extraction and classification functions and learn during training how to efficiently extract features from input images and then classify them.

### 2.5. k-Nearest Neighbors (k-NN)

k-NN is a classifier that does not belong to ANNs and is only presented for comparison with ANNs. The k-NN algorithm calculates the distance of the feature vector of the tested sample from the feature vectors of all training samples. Euclidean distance (for continuous variables) or Hamming distance (for discrete variables) are commonly used to calculate the distance. The tested sample is assigned to the class to which most of the “k” nearest training samples belong (majority voting of “k” nearest neighbors). The “k” is a pre-selected constant, and commonly used values are 3 or 5.

## 3. Results

Four types of artificial neural networks (ANNs) were tested in different configurations and their effect on the resulting classification rate was evaluated. Two experiments were designed. The aim of the first experiment was to classify the type of 2D matrix code, and the aim of the second experiment was to identify its rotation.

**Figure 10:**
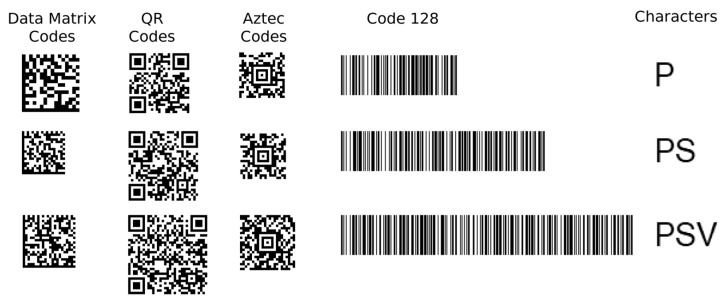
Samples from an image dataset of the first experiment.

In the first experiment, all four types of ANNs were trained on the same dataset of 1500 images (samples). These 1500 artificially generated images contained five image classes: 300 Data Matrix code images, 300 QR code images, 300 Aztec code images, 300 1D barcode images, and 300 text fragment images (“non-barcode” images). Synthetic images of 2D matrixes and 1D barcodes were generated using the open-source program qtZint and encoded 10, 20, and 30 character-long randomly generated alpha-numeric strings (therefore, within each class we had three different image sizes). Figure 10 and Table 1 show the structure of the training and testing image datasets. The testing dataset (similar to the training one) contained an additional 1500 synthetic images (File S1).

**Figure 11:**
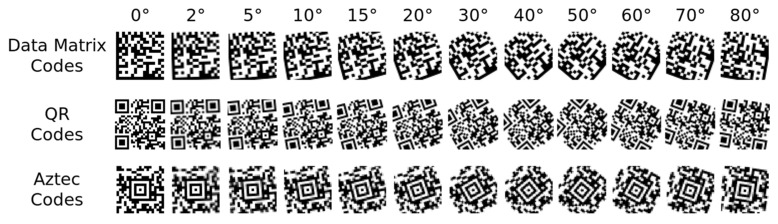
Samples from an image dataset of the second experiment.

The second experiment used 900 images from the first experiment (300 Data Matrix code, 300 QR code, and 300 Aztec code images). These images were rotated by 0, 2, 5, 10, 15, 20, 30, 40, 50, 60, 70, and 80 degrees, resulting in twelve classes according to the angle of rotation (Figure 11). Neural networks were trained to classify these twelve rotation angles.

The input of the ANNs was a 64 × 64 grayscale bitmap. Each image in the image dataset was resampled to this size. Thus, the input layer of an ANN consisted of 4096 neurons (each input neuron corresponds to one point of the input binary image (0–white point, 1–black point)). The output layer of the ANN, in the first experiment, was formed by five neurons, where each neuron corresponded to one of five classes (1: Data Matrix code, 2: QR code, 3: Aztec code, 4: Code 128 (1D barcode), 5: “non-barcode” characters). The output layer of ANNs in the second experiment was formed by twelve neurons, each neuron corresponding to one of the rotation angles (1: 0°, 2: 2°, 3: 5°, 4: 10°, 5: 15°, 6: 20°, 7: 30°, 8: 40°, 9: 50°, 10: 60°, 11: 70°, 12: 80°).

In the first experiment, each type of ANN was trained separately on the full training dataset of 1500 samples and also on its five subsets containing 150 selected samples (samples of each class were equally represented; the purpose of the test was to examine the ability of the ANN to generalise when fewer samples are available to train). As the ANN was trained individually on these five subsets of 150 samples (and then tested on the full testing dataset of 1500 samples), five classification rates were obtained. These classification rates are presented in the following tables as an interval from the worst to the best classification rate.

### 3.1. Multilayer Perceptron (MLP)

The MLP achieved a classification rate between 93.4% and 97.7% when trained with 1500 samples and then tested with another 1500 samples, as shown in Table 2.

When trained with 150 samples (selected from 1500 samples), the classification rate (depending on the selection of 150 samples) ranged from 93.9% to 95.5% for 20 neurons in the hidden layer. With 120 neurons in the hidden layer, the network converged faster (about 100 iterations compared to 440 with 20 neurons and 180 with 60 neurons) and the recognition rate was slightly better, ranging from 94.4% to 95.7%. With 720 neurons in the hidden layer, the network converged even faster (about 50 iterations, but the training time was longer) and the recognition rate dropped, ranging from 93.2% to 96.2%. Classification errors occurred mostly with Aztec codes. Adding a second hidden layer did not improve the classification rate. In fact, it made it worse.

### 3.2. Probabilistic Neural Network (PNN)

The PNN was tested for different values of the smoothing parameter sigma (σ). The choice of sigma has a significant effect on the classification rate. With a feature vector size of 4096, even small differences in sigma (to the power of σ^4096^) cause large differences in the probability of the output layer. Through testing, it was possible to find an interval of sigma values (4–5) for which PNN achieved the best classification rate (Table 3). Calculating sigma individually for individual pattern layer neurons or for pattern layer neurons belonging to the same class gave unsatisfactory results.

### 3.3. Radial Basis Function Network (RBF NN)

The RBF NN was tested in one-phase learning, where all unique samples (data points) from the training dataset were added to the hidden layer. The sigma parameter was common to all neurons of the hidden layer and was calculated as $\sigma_{1}=d_{max}/\sqrt{2M}$ (where *d*_max_ is the maximum Euclidean distance between two centers and M is the number of neurons (data points) in the hidden layer) or $\sigma_{2}=2d_{avg}$ (where *d*_avg_ is the average Euclidean distance between all centers). The weights between the hidden layer neurons and the output layer neurons were calculated analytically with the pseudo-inverse matrix calculation method.

Subsequently, the RBF NN was tested in two-phase learning, when the closest training samples were first grouped into clusters using K-Means, the centroids of the clusters were used as the parameter μ, and the parameter σ_3_ was calculated as the average distance of the samples belonging to same cluster to its centroid (if the cluster had only one sample, then σ_3_ was determined as the average distance from all the average distances of the clusters) or σ_4_ was calculated as ½ the distance of the centroid of the cluster to the nearest other centroid of another class (without ½, it was not possible to train the network). The weights between the hidden layer neurons and the output layer neurons were calculated using the error back-propagation method.

As can be seen in Table 4, the choice of the sigma parameter has a significant impact on the ability of the RBF NN to train and thus also on the classification rate.

### 3.4. Convolutional Neural Network (CNN)

The CNN was tested for different depths and different numbers of feature maps (4, 8, 16, 32). In the simplest configuration (CNN-1), the first layer was a convolutional layer (C) with a ReLU activation function, followed by a max-pooling layer (S), followed by two fully connected layers (F) with a sigmoid activation function (C1, S2, F3-4). In other configurations, even more convolutional (C) and max. pooling layers (S) were added (Table 5). The convolutional layer worked with a filter size of 3 × 3, a stride size of 1, and a padding of 1 (trials with a larger filter size of 5 × 5 showed the same or worse results). The max. pooling layer used a patch of 2 × 2 and a stride size of 2.

Similar to the MLP, all classification errors occurred mostly with Aztec codes.

As can be seen from the results of the first experiment, for each type of ANN tested, the classification rate is strongly dependent on the chosen network configuration. Similarly, the number of training samples also affects classification accuracy. A smaller number of samples (150 versus 1500) usually results in a lower classification rate. Here, the convolutional neural network seems to have the best generalisation ability, followed by the RBF neural network, followed by the multilayer perceptron, followed by the probabilistic neural network (Table 6). In addition to the ANNs, the table also shows the results of the k-NN (k-Nearest Neighbors) classifier.

The observed results of the first experiment (2D matrix code type classification) can be interpreted as follows:

- If the size of the training dataset of samples is large enough to cover a large number of variations in samples from the test dataset and/or the diversity between the training and test datasets is low, the classifier itself does not play an important role;
- Classification accuracy is not only influenced by the type of ANN, but also by correct configuration and parameterization (such as the number of layers, the number of feature maps, the number of neurons in the layers, the sigma parameter);
- The convolutional neural network achieved the best results because it is not only a classifier but also a feature extractor and is designed to work directly with images.

Table 7 shows the results obtained by the ANNs in classifying the rotation angle of 2D matrix codes in the second experiment.

The observed results of the second experiment (rotation angle classification) can be interpreted as follows:

- The number of training samples must be large enough to train the classifiers satisfactorily (the number of samples must increase as the number of output classes increases);
- A convolutional neural network with two stacked convolutional layers performed slightly better than traditional neural networks (RBF neural network and multilayer perceptron) when trained on a larger number of samples (900). However, when trained on fewer samples (90), traditional neural networks (the RBF neural network and the multilayer perceptron) outperformed convolutional networks.

A small number of training samples can disqualify convolutional neural networks which cannot effectively extract discriminative features.

## 4. Conclusions

The paper deals with the applicability of artificial neural networks for the classification of images of several types of 2D matrix codes (Data Matrix codes, QR codes, Aztec codes) and their rotation angle classification. Four types of artificial neural networks were tested in different configurations—multilayer perceptrons, probabilistic neural networks, RBF neural networks, and convolutional neural networks. Experiments showed that convolutional neural networks, trained on a sufficiently large number of representative samples, achieved the highest classification accuracy with a suitable configuration. In addition, the RBF neural network and the multilayer perceptron performed satisfactorily, even outperforming convolutional neural networks in the rotation angle classification task when trained on a smaller number of samples. Great attention must also be paid to the configuration and choice of neural network parameters, which have a significant impact on classification accuracy. Despite the fact that deep learning methods are gaining a lot of attention, traditional neural networks can still be an effective classifier, especially in cases where it is possible to efficiently extract a discriminative feature vector from the test object or where the variability between the test and training objects is low. Presently, 2D matrix codes are an established technology and can be found in a variety of places [30,31,32] and on everyday objects.

## Figures and Tables

**Table 1:** Structure of the training and testing image datasets.

Data Encoded by 2D and 1D Codes	Image Classes and Sizes				
	Data Matrix Codes	QR Codes	Aztec Codes	1D Barcode (Code 128)	Characters (A–Z)
10 alpha-numeric characters, 3 px module size	16 × 16 modules (48 × 48 px)	25 × 25 modules (75 × 75 px)	19 × 19 modules (57 × 57 px)	290 × 100 px	1 char (w1 × 32 px)
20 alpha-numeric characters, 2 px module size	18 × 18 modules (36 × 36 px)	29 × 29 modules (58 × 58 px)	19 × 19 modules (38 × 38 px)	510 × 100 px	2 chars (w2 × 32 px)
30 alpha-numeric characters, 2 px module size	22 × 22 modules (44 × 44 px)	33 × 33 modules (66 × 66 px)	23 × 23 modules (46 × 46 px)	730 × 100 px	3 chars (w3 × 32 px)

**Table 2:** Classification rates achieved by the multilayer perceptron.

Classifier	Configuration: *Number of Neurons in the Hidden Layer(s)*	Number of Training Samples	
		1500	150
Multilayer Perceptron (one hidden layer, sigmoid activation function)	20	97.3%	93.9–95.5%
	60	97.7%	94.3–95.6%
	120	97.3%	94.4–95.7%
	720	93.4%	93.2–96.2%
Multilayer Perceptron (two hidden layers, sigmoid activation function)	20, 18	67.8% *	–
	60, 45	96.5%	–
	120, 85	74.9%	–
	720, 485	76.3% *	–

(The number of neurons in the second hidden layer was set to 2/3 of the number of neurons in the first hidden layer plus the number of neurons in the output layer. Unsatisfactory results, marked with an asterisk (*), indicate that the ANN could not be trained).

**Table 3:** Classification rates achieved by the probabilistic neural network.

Classifier	Configuration: *Parameter Sigma*	Number of Training Samples	
		1500	150
Probabilistic Neural Network	sigma per sample *	64.6%	–
	sigma per class **	51.7%	–
	sigma = 3	82.7%	–
	sigma = 4	94.6%	91.7–93.7%
	sigma = 5	94.4%	91.5–92.9%
	sigma = 6	91.1%	–
	sigma = 7	86.0%	–
	sigma = 8	82.5%	–
	sigma = 15 ***	58.1%	–

*, a sigma value, calculated as half the distance between the training sample and the nearest other sample (sample with a different feature vector). **, a common sigma value for neurons in a class, calculated as half the average distance between the training samples in a class. ***, a common sigma value of 15 was chosen based on “sigma per class” as an average value calculated from half the average distance of each sample to other samples within the same class.

**Table 4:** Classification rates achieved by the radial basis function neural network.

Classifier	Configuration: *Parameter Sigma*	Number of Training Samples	
		1500	150
RBF Neural Network, one-phase learning, all unique samples (hidden neurons = 1470)	σ_1_ = 0.9	94.2%	92.2–93.0%
	σ_2_ = 66.8	20.0% *	–
	σ_2_/2 = 33.4	25.9% *	–
	σ_2_/4 = 16.7	22.1% *	–
	σ = 11	98.3%	95.4–96.2%
RBF Neural Network, two-phase learning, clustering = 5 (hidden neurons = 285)	σ = 8	97.9%	–
	σ = 9	99.7%	95.9–98.1%
	σ = 10	99.4%	–
	σ = 11	97.6%	–
	σ_3_	21.1% *	–
	σ_4_	87.9%	–
RBF Neural Network, two-phase learning, clustering = 10 (hidden neurons = 148)	σ = 8	96.9%	–
	σ = 9	98.7%	–
	σ = 10	99.3%	94.1–97.7%
	σ = 11	98.9%	–
	σ_3_	25.1% *	–
	σ_4_	89.7%	–
RBF Neural Network, two-phase learning, clustering = 15 (hidden neurons = 100)	σ = 8	96.8%	–
	σ = 9	98.1%	–
	σ = 10	99.0%	92.7–94.7%
	σ = 11	95.0%	–
	σ_3_	20.0%	–
	σ_4_	97.1%	–

* the RBF NN could not be trained (it did not converge to 0 errors on the training dataset).

**Table 5:** Classification rates achieved by the convolutional neural network. Bold is used to highlight the best result.

Classifier	Configuration: *Number of Feature Maps*	Number of Training Samples	
		1500	150
CNN-1: C1, S2, F3-4	4	96.9%	95.3–97.3%
	8	98.1%	95.7–97.4%
	16	99.3%	96.3–98.4%
	32	99.4%	97.7–98.8%
CNN-2: C1, S2, C3, S4, F5-6	4, 8	99.0%	93.9–99.2%
	8, 16	99.4%	96.3–99.3%
	16, 16	60.5%	98.1–99.1%
	16, 32	95.7%	98.7–99.7%
	32, 32	100%	98.8–99.7%
	32, 64	**100%**	**99.4–99.8%**
CNN-3: C1, S2, C3, S4, C5, S6, F7-8	4, 8, 16	99.5%	91.0–98.7%
	8, 16, 32	97.3%	91.7–99.9%
	16, 16, 16	99.3%	93.5–98.3%
	16, 32, 64	97.9%	98.1–99.6%
	32, 32, 32	99.7%	97.4–99.2%
	32, 64, 128	98.8%	97.9–99.9%
CNN-4: C1, S2, C3, S4, C5, S6, C7, S8, F9-10	Adding an additional convolutional layer (C7) and a pooling layer (S8) reduced the classification rate.		

**Table 6:** Best classification rates achieved by neural networks when trained on 150 samples.

Classifier	Configuration	Number of Training Samples	
		1500	150 (Average of Five Runs)
CNN-2: C1, S2, C3, S4, F5-6	32, 32 32, 64	100%	99.3% 99.6%
CNN-3: C1, S2, C3, S4, C5, S6, F7-8	32, 32, 32	99.7%	98.6%
RBF Neural Network	clustering = 5, σ = 9	99.7%	96.8%
Multilayer Perceptron	hidden = 60	97.7%	95.1%
Probabilistic Neural Network	sigma = 4	94.6%	92.7%
k-NN	k = 1 k = 3 k = 5	94.2% 93.6% 93.9%	92.4% 92.2% 85.4%

**Table 7:** Best classification rates achieved by neural networks for rotation angle classification.

Classifier	Configuration	Number of Training Samples	
		900	90
CNN-1: C1, C2, S3, F4-5	C1, C2: 16 feature maps C1, C2: 8 feature maps	99.3% 99.0%	72.3% 71.8%
RBF Neural Network, two-phase learning, clustering = 5	σ = 8–10	98.9%	77.7%
Multilayer Perceptron	40 neurons in hidden layer	98.4%	82.5%
CNN-1: C1, S2, F3-4	C1: 8 feature maps	98.1%	74.8%
CNN-2: C1, S2, C3, S4, F5-6	C1: 16, C3: 32 feature maps	96.3%	69.2%
Probabilistic Neural Network	sigma = 4–6	94.0%	76.8%
RBF Neural Network, one-phase learning, all unique samples	σ_1_=1.0	91.0%	75.2%
k-NN	k = 1 k = 3 k = 5	90.8% 92.7% 92.6%	74.5% 56.3% 57.8%

## Data Availability

The data presented in this study are available in supplementary material.

## Supplementary Materials

The following supporting information can be downloaded at https://www.mdpi.com/article/10.3390/jimaging9090188/s1, File S1: A ZIP file containing 3000 images of 2D matrix codes, 1D barcodes, and text fragments used to train and test the neural networks.
