# Supplementary figures and images for: Using Different Types of Artificial Neural Networks to Classify 2D Matrix Codes and Their Rotations—A Comparative Study (part 2 of 2)
Source: J Imaging. 2023 Sep 18;9(9):188. doi: 10.3390/jimaging9090188 (PMC10532761; doi:10.3390/jimaging9090188)

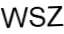

Supplement: Supplementary file 1 [file jimaging-09-00188-s001.zip › 5_03_100.png]

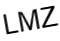

Supplement: Supplementary file 1 [file jimaging-09-00188-s001.zip › 5_03_097.png]

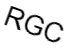

Supplement: Supplementary file 1 [file jimaging-09-00188-s001.zip › 5_03_098.png]

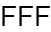

Supplement: Supplementary file 1 [file jimaging-09-00188-s001.zip › 5_03_095.png]

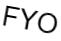

Supplement: Supplementary file 1 [file jimaging-09-00188-s001.zip › 5_03_096.png]

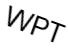

Supplement: Supplementary file 1 [file jimaging-09-00188-s001.zip › 5_03_093.png]

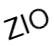

Supplement: Supplementary file 1 [file jimaging-09-00188-s001.zip › 5_03_094.png]

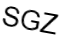

Supplement: Supplementary file 1 [file jimaging-09-00188-s001.zip › 5_03_091.png]

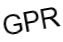

Supplement: Supplementary file 1 [file jimaging-09-00188-s001.zip › 5_03_092.png]

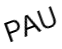

Supplement: Supplementary file 1 [file jimaging-09-00188-s001.zip › 5_03_089.png]

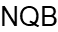

Supplement: Supplementary file 1 [file jimaging-09-00188-s001.zip › 5_03_090.png]

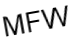

Supplement: Supplementary file 1 [file jimaging-09-00188-s001.zip › 5_03_087.png]

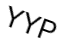

Supplement: Supplementary file 1 [file jimaging-09-00188-s001.zip › 5_03_088.png]

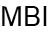

Supplement: Supplementary file 1 [file jimaging-09-00188-s001.zip › 5_03_085.png]

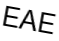

Supplement: Supplementary file 1 [file jimaging-09-00188-s001.zip › 5_03_086.png]

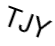

Supplement: Supplementary file 1 [file jimaging-09-00188-s001.zip › 5_03_083.png]

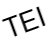

Supplement: Supplementary file 1 [file jimaging-09-00188-s001.zip › 5_03_084.png]

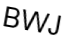

Supplement: Supplementary file 1 [file jimaging-09-00188-s001.zip › 5_03_081.png]

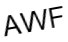

Supplement: Supplementary file 1 [file jimaging-09-00188-s001.zip › 5_03_082.png]

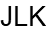

Supplement: Supplementary file 1 [file jimaging-09-00188-s001.zip › 5_03_080.png]

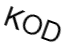

Supplement: Supplementary file 1 [file jimaging-09-00188-s001.zip › 5_03_078.png]

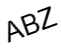

Supplement: Supplementary file 1 [file jimaging-09-00188-s001.zip › 5_03_079.png]

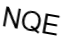

Supplement: Supplementary file 1 [file jimaging-09-00188-s001.zip › 5_03_076.png]

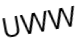

Supplement: Supplementary file 1 [file jimaging-09-00188-s001.zip › 5_03_077.png]

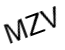

Supplement: Supplementary file 1 [file jimaging-09-00188-s001.zip › 5_03_074.png]

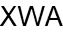

Supplement: Supplementary file 1 [file jimaging-09-00188-s001.zip › 5_03_075.png]

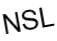

Supplement: Supplementary file 1 [file jimaging-09-00188-s001.zip › 5_03_072.png]

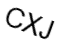

Supplement: Supplementary file 1 [file jimaging-09-00188-s001.zip › 5_03_073.png]

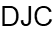

Supplement: Supplementary file 1 [file jimaging-09-00188-s001.zip › 5_03_070.png]

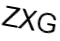

Supplement: Supplementary file 1 [file jimaging-09-00188-s001.zip › 5_03_071.png]

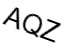

Supplement: Supplementary file 1 [file jimaging-09-00188-s001.zip › 5_03_068.png]

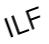

Supplement: Supplementary file 1 [file jimaging-09-00188-s001.zip › 5_03_069.png]

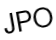

Supplement: Supplementary file 1 [file jimaging-09-00188-s001.zip › 5_03_067.png]

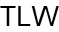

Supplement: Supplementary file 1 [file jimaging-09-00188-s001.zip › 5_03_065.png]

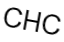

Supplement: Supplementary file 1 [file jimaging-09-00188-s001.zip › 5_03_066.png]

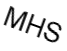

Supplement: Supplementary file 1 [file jimaging-09-00188-s001.zip › 5_03_063.png]

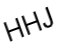

Supplement: Supplementary file 1 [file jimaging-09-00188-s001.zip › 5_03_064.png]

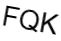

Supplement: Supplementary file 1 [file jimaging-09-00188-s001.zip › 5_03_061.png]

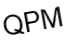

Supplement: Supplementary file 1 [file jimaging-09-00188-s001.zip › 5_03_062.png]

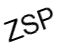

Supplement: Supplementary file 1 [file jimaging-09-00188-s001.zip › 5_03_059.png]

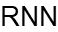

Supplement: Supplementary file 1 [file jimaging-09-00188-s001.zip › 5_03_060.png]

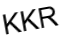

Supplement: Supplementary file 1 [file jimaging-09-00188-s001.zip › 5_03_057.png]

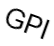

Supplement: Supplementary file 1 [file jimaging-09-00188-s001.zip › 5_03_058.png]

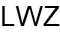

Supplement: Supplementary file 1 [file jimaging-09-00188-s001.zip › 5_03_055.png]

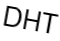

Supplement: Supplementary file 1 [file jimaging-09-00188-s001.zip › 5_03_056.png]

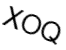

Supplement: Supplementary file 1 [file jimaging-09-00188-s001.zip › 5_03_053.png]

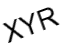

Supplement: Supplementary file 1 [file jimaging-09-00188-s001.zip › 5_03_054.png]

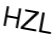

Supplement: Supplementary file 1 [file jimaging-09-00188-s001.zip › 5_03_051.png]

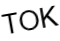

Supplement: Supplementary file 1 [file jimaging-09-00188-s001.zip › 5_03_052.png]

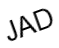

Supplement: Supplementary file 1 [file jimaging-09-00188-s001.zip › 5_03_049.png]

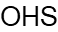

Supplement: Supplementary file 1 [file jimaging-09-00188-s001.zip › 5_03_050.png]

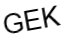

Supplement: Supplementary file 1 [file jimaging-09-00188-s001.zip › 5_03_047.png]

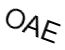

Supplement: Supplementary file 1 [file jimaging-09-00188-s001.zip › 5_03_048.png]

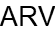

Supplement: Supplementary file 1 [file jimaging-09-00188-s001.zip › 5_03_045.png]

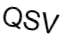

Supplement: Supplementary file 1 [file jimaging-09-00188-s001.zip › 5_03_046.png]

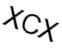

Supplement: Supplementary file 1 [file jimaging-09-00188-s001.zip › 5_03_043.png]

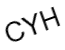

Supplement: Supplementary file 1 [file jimaging-09-00188-s001.zip › 5_03_044.png]

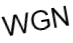

Supplement: Supplementary file 1 [file jimaging-09-00188-s001.zip › 5_03_042.png]

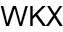

Supplement: Supplementary file 1 [file jimaging-09-00188-s001.zip › 5_03_040.png]

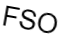

Supplement: Supplementary file 1 [file jimaging-09-00188-s001.zip › 5_03_041.png]

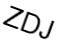

Supplement: Supplementary file 1 [file jimaging-09-00188-s001.zip › 5_03_038.png]

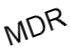

Supplement: Supplementary file 1 [file jimaging-09-00188-s001.zip › 5_03_039.png]

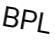

Supplement: Supplementary file 1 [file jimaging-09-00188-s001.zip › 5_03_036.png]

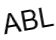

Supplement: Supplementary file 1 [file jimaging-09-00188-s001.zip › 5_03_037.png]

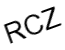

Supplement: Supplementary file 1 [file jimaging-09-00188-s001.zip › 5_03_034.png]

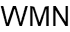

Supplement: Supplementary file 1 [file jimaging-09-00188-s001.zip › 5_03_035.png]

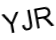

Supplement: Supplementary file 1 [file jimaging-09-00188-s001.zip › 5_03_032.png]

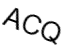

Supplement: Supplementary file 1 [file jimaging-09-00188-s001.zip › 5_03_033.png]

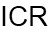

Supplement: Supplementary file 1 [file jimaging-09-00188-s001.zip › 5_03_030.png]

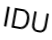

Supplement: Supplementary file 1 [file jimaging-09-00188-s001.zip › 5_03_031.png]

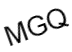

Supplement: Supplementary file 1 [file jimaging-09-00188-s001.zip › 5_03_029.png]

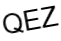

Supplement: Supplementary file 1 [file jimaging-09-00188-s001.zip › 5_03_027.png]

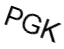

Supplement: Supplementary file 1 [file jimaging-09-00188-s001.zip › 5_03_028.png]

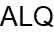

Supplement: Supplementary file 1 [file jimaging-09-00188-s001.zip › 5_03_025.png]

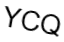

Supplement: Supplementary file 1 [file jimaging-09-00188-s001.zip › 5_03_026.png]

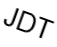

Supplement: Supplementary file 1 [file jimaging-09-00188-s001.zip › 5_03_023.png]

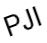

Supplement: Supplementary file 1 [file jimaging-09-00188-s001.zip › 5_03_024.png]

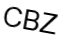

Supplement: Supplementary file 1 [file jimaging-09-00188-s001.zip › 5_03_021.png]

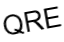

Supplement: Supplementary file 1 [file jimaging-09-00188-s001.zip › 5_03_022.png]

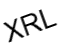

Supplement: Supplementary file 1 [file jimaging-09-00188-s001.zip › 5_03_019.png]

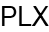

Supplement: Supplementary file 1 [file jimaging-09-00188-s001.zip › 5_03_020.png]

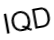

Supplement: Supplementary file 1 [file jimaging-09-00188-s001.zip › 5_03_017.png]

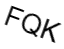

Supplement: Supplementary file 1 [file jimaging-09-00188-s001.zip › 5_03_018.png]

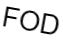

Supplement: Supplementary file 1 [file jimaging-09-00188-s001.zip › 5_03_016.png]

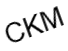

Supplement: Supplementary file 1 [file jimaging-09-00188-s001.zip › 5_03_014.png]

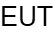

Supplement: Supplementary file 1 [file jimaging-09-00188-s001.zip › 5_03_015.png]

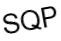

Supplement: Supplementary file 1 [file jimaging-09-00188-s001.zip › 5_03_012.png]

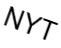

Supplement: Supplementary file 1 [file jimaging-09-00188-s001.zip › 5_03_013.png]

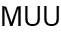

Supplement: Supplementary file 1 [file jimaging-09-00188-s001.zip › 5_03_010.png]

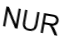

Supplement: Supplementary file 1 [file jimaging-09-00188-s001.zip › 5_03_011.png]

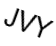

Supplement: Supplementary file 1 [file jimaging-09-00188-s001.zip › 5_03_008.png]

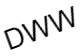

Supplement: Supplementary file 1 [file jimaging-09-00188-s001.zip › 5_03_009.png]

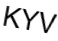

Supplement: Supplementary file 1 [file jimaging-09-00188-s001.zip › 5_03_006.png]

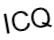

Supplement: Supplementary file 1 [file jimaging-09-00188-s001.zip › 5_03_007.png]

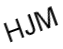

Supplement: Supplementary file 1 [file jimaging-09-00188-s001.zip › 5_03_004.png]

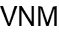

Supplement: Supplementary file 1 [file jimaging-09-00188-s001.zip › 5_03_005.png]
