# Supplementary figures and images for: Using Different Types of Artificial Neural Networks to Classify 2D Matrix Codes and Their Rotations—A Comparative Study (part 1 of 2)
Source: J Imaging. 2023 Sep 18;9(9):188. doi: 10.3390/jimaging9090188 (PMC10532761; doi:10.3390/jimaging9090188)

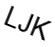

Supplement: Supplementary file 1 [file jimaging-09-00188-s001.zip › 5_03_198.png]

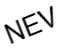

Supplement: Supplementary file 1 [file jimaging-09-00188-s001.zip › 5_03_199.png]

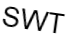

Supplement: Supplementary file 1 [file jimaging-09-00188-s001.zip › 5_03_196.png]

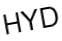

Supplement: Supplementary file 1 [file jimaging-09-00188-s001.zip › 5_03_197.png]

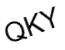

Supplement: Supplementary file 1 [file jimaging-09-00188-s001.zip › 5_03_194.png]

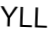

Supplement: Supplementary file 1 [file jimaging-09-00188-s001.zip › 5_03_195.png]

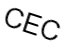

Supplement: Supplementary file 1 [file jimaging-09-00188-s001.zip › 5_03_193.png]

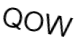

Supplement: Supplementary file 1 [file jimaging-09-00188-s001.zip › 5_03_191.png]

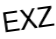

Supplement: Supplementary file 1 [file jimaging-09-00188-s001.zip › 5_03_192.png]

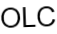

Supplement: Supplementary file 1 [file jimaging-09-00188-s001.zip › 5_03_190.png]

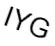

Supplement: Supplementary file 1 [file jimaging-09-00188-s001.zip › 5_03_188.png]

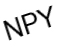

Supplement: Supplementary file 1 [file jimaging-09-00188-s001.zip › 5_03_189.png]

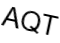

Supplement: Supplementary file 1 [file jimaging-09-00188-s001.zip › 5_03_186.png]

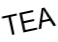

Supplement: Supplementary file 1 [file jimaging-09-00188-s001.zip › 5_03_187.png]

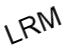

Supplement: Supplementary file 1 [file jimaging-09-00188-s001.zip › 5_03_184.png]

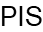

Supplement: Supplementary file 1 [file jimaging-09-00188-s001.zip › 5_03_185.png]

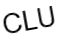

Supplement: Supplementary file 1 [file jimaging-09-00188-s001.zip › 5_03_182.png]

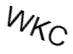

Supplement: Supplementary file 1 [file jimaging-09-00188-s001.zip › 5_03_183.png]

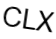

Supplement: Supplementary file 1 [file jimaging-09-00188-s001.zip › 5_03_181.png]

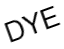

Supplement: Supplementary file 1 [file jimaging-09-00188-s001.zip › 5_03_179.png]

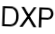

Supplement: Supplementary file 1 [file jimaging-09-00188-s001.zip › 5_03_180.png]

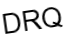

Supplement: Supplementary file 1 [file jimaging-09-00188-s001.zip › 5_03_177.png]

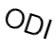

Supplement: Supplementary file 1 [file jimaging-09-00188-s001.zip › 5_03_178.png]

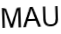

Supplement: Supplementary file 1 [file jimaging-09-00188-s001.zip › 5_03_175.png]

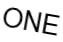

Supplement: Supplementary file 1 [file jimaging-09-00188-s001.zip › 5_03_176.png]

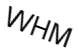

Supplement: Supplementary file 1 [file jimaging-09-00188-s001.zip › 5_03_173.png]

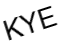

Supplement: Supplementary file 1 [file jimaging-09-00188-s001.zip › 5_03_174.png]

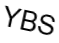

Supplement: Supplementary file 1 [file jimaging-09-00188-s001.zip › 5_03_171.png]

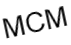

Supplement: Supplementary file 1 [file jimaging-09-00188-s001.zip › 5_03_172.png]

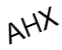

Supplement: Supplementary file 1 [file jimaging-09-00188-s001.zip › 5_03_169.png]

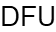

Supplement: Supplementary file 1 [file jimaging-09-00188-s001.zip › 5_03_170.png]

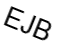

Supplement: Supplementary file 1 [file jimaging-09-00188-s001.zip › 5_03_168.png]

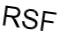

Supplement: Supplementary file 1 [file jimaging-09-00188-s001.zip › 5_03_166.png]

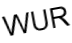

Supplement: Supplementary file 1 [file jimaging-09-00188-s001.zip › 5_03_167.png]

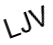

Supplement: Supplementary file 1 [file jimaging-09-00188-s001.zip › 5_03_164.png]

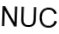

Supplement: Supplementary file 1 [file jimaging-09-00188-s001.zip › 5_03_165.png]

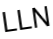

Supplement: Supplementary file 1 [file jimaging-09-00188-s001.zip › 5_03_162.png]

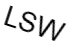

Supplement: Supplementary file 1 [file jimaging-09-00188-s001.zip › 5_03_163.png]

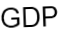

Supplement: Supplementary file 1 [file jimaging-09-00188-s001.zip › 5_03_160.png]

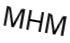

Supplement: Supplementary file 1 [file jimaging-09-00188-s001.zip › 5_03_161.png]

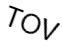

Supplement: Supplementary file 1 [file jimaging-09-00188-s001.zip › 5_03_158.png]

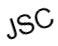

Supplement: Supplementary file 1 [file jimaging-09-00188-s001.zip › 5_03_159.png]

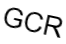

Supplement: Supplementary file 1 [file jimaging-09-00188-s001.zip › 5_03_156.png]

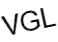

Supplement: Supplementary file 1 [file jimaging-09-00188-s001.zip › 5_03_157.png]

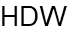

Supplement: Supplementary file 1 [file jimaging-09-00188-s001.zip › 5_03_155.png]

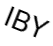

Supplement: Supplementary file 1 [file jimaging-09-00188-s001.zip › 5_03_153.png]

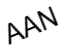

Supplement: Supplementary file 1 [file jimaging-09-00188-s001.zip › 5_03_154.png]

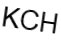

Supplement: Supplementary file 1 [file jimaging-09-00188-s001.zip › 5_03_151.png]

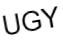

Supplement: Supplementary file 1 [file jimaging-09-00188-s001.zip › 5_03_152.png]

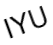

Supplement: Supplementary file 1 [file jimaging-09-00188-s001.zip › 5_03_149.png]

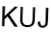

Supplement: Supplementary file 1 [file jimaging-09-00188-s001.zip › 5_03_150.png]

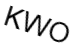

Supplement: Supplementary file 1 [file jimaging-09-00188-s001.zip › 5_03_148.png]

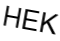

Supplement: Supplementary file 1 [file jimaging-09-00188-s001.zip › 5_03_146.png]

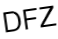

Supplement: Supplementary file 1 [file jimaging-09-00188-s001.zip › 5_03_147.png]

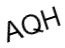

Supplement: Supplementary file 1 [file jimaging-09-00188-s001.zip › 5_03_144.png]

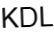

Supplement: Supplementary file 1 [file jimaging-09-00188-s001.zip › 5_03_145.png]

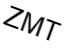

Supplement: Supplementary file 1 [file jimaging-09-00188-s001.zip › 5_03_143.png]

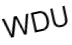

Supplement: Supplementary file 1 [file jimaging-09-00188-s001.zip › 5_03_142.png]

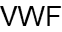

Supplement: Supplementary file 1 [file jimaging-09-00188-s001.zip › 5_03_140.png]

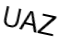

Supplement: Supplementary file 1 [file jimaging-09-00188-s001.zip › 5_03_141.png]

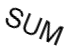

Supplement: Supplementary file 1 [file jimaging-09-00188-s001.zip › 5_03_138.png]

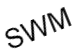

Supplement: Supplementary file 1 [file jimaging-09-00188-s001.zip › 5_03_139.png]

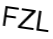

Supplement: Supplementary file 1 [file jimaging-09-00188-s001.zip › 5_03_136.png]

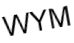

Supplement: Supplementary file 1 [file jimaging-09-00188-s001.zip › 5_03_137.png]

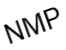

Supplement: Supplementary file 1 [file jimaging-09-00188-s001.zip › 5_03_134.png]

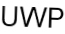

Supplement: Supplementary file 1 [file jimaging-09-00188-s001.zip › 5_03_135.png]

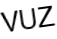

Supplement: Supplementary file 1 [file jimaging-09-00188-s001.zip › 5_03_132.png]

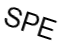

Supplement: Supplementary file 1 [file jimaging-09-00188-s001.zip › 5_03_133.png]

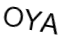

Supplement: Supplementary file 1 [file jimaging-09-00188-s001.zip › 5_03_131.png]

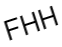

Supplement: Supplementary file 1 [file jimaging-09-00188-s001.zip › 5_03_129.png]

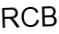

Supplement: Supplementary file 1 [file jimaging-09-00188-s001.zip › 5_03_130.png]

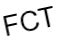

Supplement: Supplementary file 1 [file jimaging-09-00188-s001.zip › 5_03_127.png]

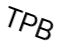

Supplement: Supplementary file 1 [file jimaging-09-00188-s001.zip › 5_03_128.png]

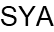

Supplement: Supplementary file 1 [file jimaging-09-00188-s001.zip › 5_03_125.png]

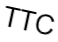

Supplement: Supplementary file 1 [file jimaging-09-00188-s001.zip › 5_03_126.png]

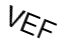

Supplement: Supplementary file 1 [file jimaging-09-00188-s001.zip › 5_03_123.png]

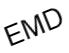

Supplement: Supplementary file 1 [file jimaging-09-00188-s001.zip › 5_03_124.png]

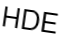

Supplement: Supplementary file 1 [file jimaging-09-00188-s001.zip › 5_03_121.png]

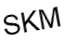

Supplement: Supplementary file 1 [file jimaging-09-00188-s001.zip › 5_03_122.png]

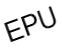

Supplement: Supplementary file 1 [file jimaging-09-00188-s001.zip › 5_03_119.png]

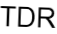

Supplement: Supplementary file 1 [file jimaging-09-00188-s001.zip › 5_03_120.png]

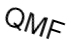

Supplement: Supplementary file 1 [file jimaging-09-00188-s001.zip › 5_03_118.png]

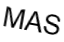

Supplement: Supplementary file 1 [file jimaging-09-00188-s001.zip › 5_03_116.png]

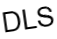

Supplement: Supplementary file 1 [file jimaging-09-00188-s001.zip › 5_03_117.png]

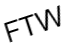

Supplement: Supplementary file 1 [file jimaging-09-00188-s001.zip › 5_03_114.png]

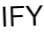

Supplement: Supplementary file 1 [file jimaging-09-00188-s001.zip › 5_03_115.png]

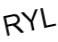

Supplement: Supplementary file 1 [file jimaging-09-00188-s001.zip › 5_03_112.png]

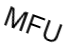

Supplement: Supplementary file 1 [file jimaging-09-00188-s001.zip › 5_03_113.png]

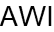

Supplement: Supplementary file 1 [file jimaging-09-00188-s001.zip › 5_03_110.png]

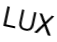

Supplement: Supplementary file 1 [file jimaging-09-00188-s001.zip › 5_03_111.png]

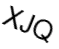

Supplement: Supplementary file 1 [file jimaging-09-00188-s001.zip › 5_03_108.png]

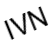

Supplement: Supplementary file 1 [file jimaging-09-00188-s001.zip › 5_03_109.png]

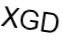

Supplement: Supplementary file 1 [file jimaging-09-00188-s001.zip › 5_03_106.png]

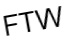

Supplement: Supplementary file 1 [file jimaging-09-00188-s001.zip › 5_03_107.png]

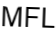

Supplement: Supplementary file 1 [file jimaging-09-00188-s001.zip › 5_03_105.png]

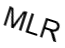

Supplement: Supplementary file 1 [file jimaging-09-00188-s001.zip › 5_03_103.png]

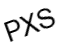

Supplement: Supplementary file 1 [file jimaging-09-00188-s001.zip › 5_03_104.png]

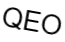

Supplement: Supplementary file 1 [file jimaging-09-00188-s001.zip › 5_03_101.png]

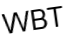

Supplement: Supplementary file 1 [file jimaging-09-00188-s001.zip › 5_03_102.png]

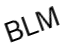

Supplement: Supplementary file 1 [file jimaging-09-00188-s001.zip › 5_03_099.png]
